# Challenges to patient centredness – a comparison of patient and doctor experiences from primary care

**DOI:** 10.1186/s12875-019-0959-y

**Published:** 2019-06-15

**Authors:** Helene Bodegård, Gert Helgesson, Niklas Juth, Daniel Olsson, Niels Lynøe

**Affiliations:** 1 Stockholm Centre for Healthcare Ethics, Department of Learning, Informatics, Management and Ethics (LIME), Karolinska Institutet, Tomtebodavägen 18 A, 171 77, Stockholm, Sweden; 2 Unit of Medical Statistics, Department of Learning, Informatics, Management and Ethics (LIME), Karolinska Institutet, Tomtebodavägen 18A, 171 77, Stockholm, Sweden

**Keywords:** Patient-centred care, Communication skills, Patient-doctor communication, Shared decision making, Patient feedback, Reason for encounter, Reason for visit, General practice, Family practice

## Abstract

**Background:**

We designed this observational study to investigate the level of patients’ and doctors’ ratings of patient-centred aspects of the primary care consultation.

**Methods:**

Questionnaire study with patients and doctors. Consecutive patients in a primary care setting and 16 doctors responding post visit. Results are presented as proportions with 95% confidence intervals.

**Results:**

411 questionnaires, 223 from patients and 188 from doctors, covered 251 consultations. Both patients and doctors gave the highest possible estimations on the aspects of patient-centred communication and satisfaction less frequently when the patient had other reasons for visit than purely somatic. Unlike the doctors’ estimations, the frequency of highest possible estimations in patient responses dropped if the patients had two to six reasons for visit rather than one. Among the six patient-centred aspects, both patients and doctors gave the highest possible estimation least frequently on the aspect of *shared decision-making.*

**Conclusion:**

The results suggest that the nature of the reason, as well as the number of reasons for visit, interferes with the doctors’ level of patient-centred communication. Our results furthermore confirm the findings of previous studies that doctors insufficiently involve patients in their care.

**Electronic supplementary material:**

The online version of this article (10.1186/s12875-019-0959-y) contains supplementary material, which is available to authorized users.

## Background

Many countries and international organisations, such as OECD and WHO, have chosen patient centredness as the approach to ensure that the patients’ needs, values, and preferences are appropriately considered in the health care meetings [1, 2]. Whereas most OECD countries show progress in implementing patient-centred care, no country performs in the top group on all indicators in cross-country comparison [3]. Patient-centred consultation methodologies vary, but reoccurring components can be identified in the literature, such as the *patient’s narrative* and *collaboration* [4]. The narrative component can be described as the patient’s spontaneous description of their problem, including their thoughts, feelings, and experiences [5]. The patient’s agenda, signified as ideas, concerns, and expectations, is an example of narrative aspects found to be central in understanding the patient and their perception of their problem(s) [6–8]. Enabling the patient’s narrative gives the doctor an understanding of which questions need to be answered, in order to give the patient the information required to take part in the decisions about their own care. This is referred to as *shared decision-making* [9, 10]*.*

Numerous studies analysing the ways doctors and patients communicate have suggested that in consultations where patient-centred components are missing, relevant information is often missed too [11]. Unnecessary referrals and prescriptions of medication increase, and the patients are less content [6, 9, 12–14]. The patient’s mental health, adherence to treatment, and recovery are other health-related outcomes that increase with a patient-centred consultation [6, 9, 13, 14]. Furthermore, patients in primary care, and especially vulnerable patients (psychosocially or in terms of not feeling well), want a patient-centred approach [15].

The implementation of patient centredness has, however, been limited in Sweden. For instance, it has been shown that patients in Sweden are not sufficiently informed or involved in the decision-making process [1, 16]. These findings are consistent with European comparisons [17]. A new Patient Act was introduced in Sweden in 2015 with the main purpose to increase focus on patient autonomy and strengthen patients’ involvement in their own care [18].

Some associations have been found between individual characteristics of the patients and doctors and the level of patient centredness, such as the gender of patients and doctors, the patients’ age, and the doctors’ work experience [19–21]. However, more information on factors that interfere with the doctors’ inclination to be patient centred is needed. In a Swedish primary care centre, the patient’s reason for the encounter is often specified in the booking and medical chart, recorded in short terms such as: “chest pain”, “sick-leave certificate”, or “depression?”. One of the first tasks in the consultation procedure is for the doctor to understand this reason for the visit, and the patient’s agenda can be seen as the extended version. Providing the patient with an opportunity to make clear their reason(s) for the encounter, regardless of what is written in the booking or previously agreed on, can be seen as a prerequisite for it to be patient centred and for the doctor to know how to continue in the patient interview [12]. Comparisons between patients’ and doctors’ descriptions of the patient’s reason for encounter have previously been made, showing a 75% concordance between patient and doctor responses [22]. However, there is a scarcity of studies exploring the patient’s reason for encounter and its effect on consultation and outcome measures.

The main purpose of the present study is to investigate how patients and doctors respectively perceive patient-centred aspects in their encounters and what affects this perception.

## Methods

### Setting and participants

A questionnaire survey was distributed to and responded by patients and doctors at a primary care centre in a suburb in the northern parts of Stockholm (Sweden) during one week in February and one week in May 2016. The weeks were chosen to meet the requests of the primary care centre and the research period was divided in two to ease the administrative burden of the participating doctors. The general practice in this study serves a population of 17,500 patients. Both elderly and patients born abroad are represented to a greater extent than in Sweden in general. The practice is active in research and training of medical students. All patients aged 18 and above who attended the primary care centre during the research period, and doctors in service, were asked to participate in the survey. They had 15 to 30 min for each consultation, depending on the character of the medical problem. Contrary to instruction, seven children (and/or parents to children) completed the questionnaire. These questionnaires have *not* been excluded.

Initially the study intended to include all health care providers in duty. When noted that a large proportion of questionnaires had to be excluded due to patient questionnaires being filled out with the assistance of a health care provider, only a few questionnaires from other professionals than doctors remained. Therefore only responses from doctors, patients to doctors, and unmatched patients will be presented.

### The questionnaire

The questionnaire was developed on the basis of the main items in the patient-centred consultation model taught to students and supervisors in the physicians program at Karolinska Institutet. In the model (see Additional file 1) the items are operationalised chronologically in three parts: the patient’s part, the doctor’s part and the shared part [23–27]. There were two versions of the questionnaire: one to patients and one to physicians.

The questionnaire started out with two free-text response questions on (a) the main reason for the patient to attend the clinic (in the doctor’s version how the doctor perceived this reason) and (b) if the patient had additional reasons to consult the doctor, and in that case which these additional reasons were. There were seven statements with fixed response options and a free-text commentary possibility at the end.

The statements to patients were mirrored in the questionnaire to healthcare providers. For instance, in the patient questionnaire it was stated: “You got your questions answered.” In the questionnaire to the doctors the corresponding statement was “The patient got their questions answered” (see Table 1).

**Table 1:** The statements in the patient and doctor questionnaires

Statement	Patient questionnaire	Doctor questionnaire
1	You described your own ideas regarding your ailment/problem, your concerns and what you wished for/expected of the visit	The patient described their own ideas regarding their ailment/problem, their concerns and what he/she wished for/expected of the visit
2	The caregiver listened to you without interrupting	I listened to the patient without interrupting
3	You experienced that you were taken seriously when you told about your ailments/problems	I took what the patient told me seriously
4	You were informed about the caregiver’s assessment on your need for measures to be taken	The patient was informed about my assessment on the need for measures to be taken
5	You got your questions answered	The patient got their questions answered
6	You were invited to participate in the decision-making regarding your care (examination/treatment)	The patient was invited to participate in the decision-making regarding their examination/treatment
7	You are satisfied with your visit	I am satisfied with my performance during the visit

The response options to statements 1–7 were given on a four-point Likert scale from “I agree completely”, “I agree to a large extent”, and “I disagree to a large extent” to “I disagree completely”. In addition, the survey contained background questions. Background questions for patients included age, gender, country of birth (Sweden, other European country or outside of Europe), and education (primary school, secondary school/gymnasium, professional school or university). The doctor questionnaire included questions about gender and profession. (The complete questionnaire versions are provided in Additional files 2 and 3).

The questionnaire was piloted among 95 patients and 16 doctors during two days. This led to minor alterations of the questions and a joint decision with the doctors on method of distribution. Prior to the research period, the doctors at the primary care centre were informed about the data collection and answered a base-line survey with questions about (1) their conception of patient-centred care, (2) their self-rated level of experience of working in a patient-centred manner, and (3) their perceptions on patient participation. Question (1) and (3) were answered by supplying free-text responses and question (2) was answered by responding with one of the following alternatives: “I have large experience”, “I have little experience”, “I don’t have any experience” and “I don’t know what patient-centred care is”.

Neither the base-line survey nor the questionnaires filled out by the physicians were coded in such a way that the responses could be tied to individual respondents. To further secure the participants’ anonymity, the doctors did not state their age or level of experience in the questionnaires.

### Data collection

All potential participants were informed about the purpose of the study, that the survey was anonymous, and that no doctor would have access to the material. If verbally consenting to participate, patients and doctors completed the questionnaire; those who declined participation opted out by not filling out the questionnaire. Patients were informed and offered by the doctor at the end of consultation to answer the questionnaire in the waiting room after the visit. The participating doctors answered a similar questionnaire regarding the same consultation. The questionnaires were coded with numbers so that they could be matched pairwise in order to get the two perspectives of the patient and the doctor on the same consultation.

If the doctor had failed to provide the patient with a questionnaire, the patient was provided with one from a researcher in the waiting room. When completed, the patient questionnaires were collected by researchers in the waiting rooms. The doctors’ questionnaires were collected at the end of each day.

### Data analysis

When dividing the patients into four age groups, we wanted four equally sized groups. We therefore let the group size guide the cut-off for each group. When categorising the patients’ reasons for visit, we let the patients’ response guide the choice of category when possible. We let the doctors’ responses help to interpret or add to the patients’ responses. If the patient had not responded at all, we based the choice of reason for visit category on the doctor’s response. The responses were coded into five groups: (I) Somatic illness, (II) Mental illness, (III) Certification/ Administrative, (IV) Mixed (two or all three of the above), or (V) Unspecific.

Data was analysed using both descriptive statistics (proportions with 95% confidence interval and compared proportions using VassarStats) and hypothesis testing (Chi-2 test). When responses to questions 1–7 were analysed, the responses were dichotomised into two major groups: “I agree completely” and “I do not agree completely”, the former including the answer “I agree completely” and the latter including the three remaining response options [1]. As the physicians participating in the study did not agree to their questionnaires being coded to an individual physician, intra class correlation (ICC), identifying potential clustering at the individual physician level, could not be estimated from the study. For those results where significant differences were found using CHI-2 test, additional sensitivity analyses were performed. These analyses presented F-tests assuming independence of observations from the patient questionnaires and a known ICC of 0.10 for the observations from the physician questionnaires [28]. It was also assumed that each physician had an equal number of patient visits. The highest assumed ICC where the comparison is still significant at the 0.05 level was also reported. The patients’ responses are presented as proportions with 95% confidence intervals (CI).

The free-text responses at the end of the questionnaire and the base-line surveys were categorised with the main purpose of facilitating the interpretation of the quantitative material.

Our study aimed to explore the following research questions: (1) Is there any of the patient factors, background variables or reasons for visit that poses a challenge to the doctors’ level of patient-centred communication? (2) Do patients and doctors perceive the occurrence of the aspects similarly? (3) Is there any of the aspects that could be identified as specifically problematic and (4) Do patients and doctors perceive the patients reason for visit in the same way?

## Results

The 16 participating doctors, eleven female and five male, had a professional level that ranged from internship to specialists in general medicine. They rated their experience of working in a patient-centred manner as large (nine), limited (five) or non-existing (two). The background variables of the participating patients are presented in Table 2.

**Table 2:** Background variables of patients

	Females (%)	Males (%)	All
	*n* = 143 (64.7)	*n* = 78 (35.3)	*n* = 221
Age			
0–32	36 (25.4)	16 (21.3)	52 (23.9)
33–49	35 (24.6)	19 (25.3)	54 (24.9)
50–67	33 (23.2)	22 (29.3)	55 (25.3)
68–99	38 (26.8)	18 (24.0)	56 (25.8)
Origin			
Sweden	92 (65.7)	49 (64.5)	141 (65.3)
Europe	17 (12.1)	8 (10.5)	25 (11.6)
Outside Europe	31 (22.1)	19 (25.0)	50 (23.1)
Education			
Primary school	29 (21.6)	8 (12.8)	37 (18.9)
Secondary school/ Gymnasium	12 (9.0)	7 (11.3)	19 (9.7)
Professional school	49 (36.6)	19 (30.6)	68 (34.7)
University	44 (32.8)	28 (45.2)	72 (36.7)
Reason for encounter			
Somatic reason	100 (75.8)	61 (84.7)	161 (78.9)
Mental reason	3 (2.3)	0 (0.0)	3 (1.5)
Certifications/ administrative	11 (8.3)	2 (2.8)	13 (6.4)
Mixed	15 (11.4)	5 (6.9)	20 (9.8)
Unspecific reason	3 (2.3)	4 (5.6)	7 (3.4)
No of reasons for Encounter			
One	102 (77.3)	57 (79.2)	159 (77.9)
Two – six	30 (22.7)	15 (20.8)	45 (22.1)

The results are presented as numbers and percent (in brackets) of the participating patients. Responses about reason for visit from unmatched patient- and matched patient/doctor questionnaires only. The answers of the two patients that did not state their gender are not included in this table. Internal number of drop-outs varied between 2 and 27

Some of the patients declined or had difficulties participating in the study. Of those presenting a reason, language difficulties (three patients), severe illness (one patient) and anger with the visit in question was mentioned. Among the doctors, one person declined participation. Among the doctors participating, questionnaires were not always completed for all consultations. In total, 411 questionnaires were registered, consisting of 223 patient questionnaires and 188 doctor questionnaires (160 matched pairs of patient and doctor questionnaires, 63 unmatched patient questionnaires and 28 unmatched doctor questionnaires), covering 251 consultations.

Reasons for encounter were reported for 231 consultations (92%). The response rate on reasons for encounter was 53.8% for the patients and 93.1% for the doctors. Reasons for encounter were reported by *both* patients and doctors in 40% of the 160 pairs of matched questionnaires, thus covering both perspectives on 64 consultations.

### Main results

**Fig. 1:**
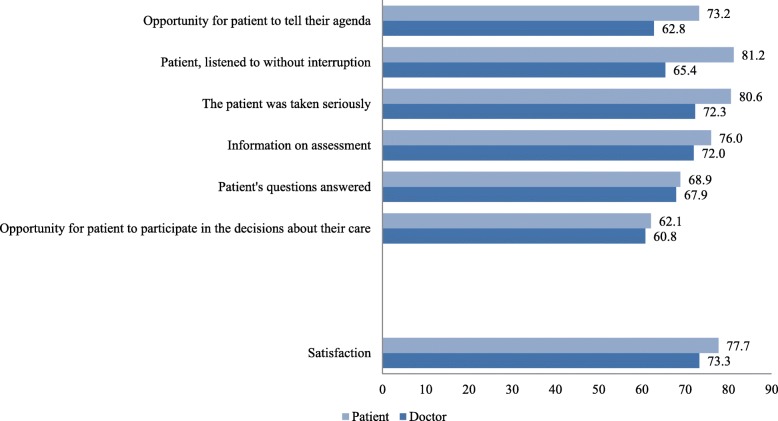
Patient and doctor ratings on each patient-centered aspect and satisfaction in general practice consultations. Percentage for patient and doctor responses with the highest possible estimation. The internal number of drop-outs varied between 0–12 for patients and 0–2 for doctors

The most common response among patients as well as physicians to all seven statements in the questionnaire was “I agree completely”. Patients gave the highest possible estimation (I agree completely) most frequently for the aspects of *being listened to without interruption* [81.2% (Confidence interval, CI: 76.1–86.3)] and *being taken seriously* [80.6%; (CI: 75.4–85.8)]. The doctors generally gave this rating less frequently than the patients. The aspect *Patient’s opportunity to participate in the decisions concerning their ongoing care* was given the lowest proportion of the highest possible estimation by both patients [62.1% (CI: 55.6–68.6)] and doctors (60.8%). For an overview of patient and doctor estimations of six patient-centred aspects and overall satisfaction, see Table 3 and Fig. 1.

**Table 3:** Differences between patient and doctor ratings on patient-centred aspects in general practice consultations

Patient-centred aspect	Total no of patient responses. (Percentage with highest possible estimations)	Total no of doctor responses. (Percentage with highest possible estimations)	Difference between patients and doctors *p*-value CHI2-test	Difference between patients and doctors *p*-value F-test ICC (doctors) = 0.1	Maximum ICC (doctors) for *p*-value ≤0.05 F-test
*1. The patient was provided an opportunity to describe their discomforts, ideas, concerns and expectations*	220 (73.2)	188 (62.8)	0.024	0.131	0.02
*2. The doctor listened without interrupting*	223 (81.2)	188 (65.4)	< 0.001	0.014	0.19
*3. The patient was taken seriously*	222 (80.6)	188 (72.3)	0.047	0.185	0.00

Total number of patient and doctor responses and percentage of patient and doctor responses with the highest possible estimation on the patient-centred aspects where there was a difference between the patients’ and doctors’ ratings. There were no internal drop outs among the doctors responses. The number of drop-outs varied between 0 and 3 for the patients

### Comparison between subgroups

The doctors’ estimations of all six patient-centred aspects and overall satisfaction were given the highest possible rating (“I agree completely”) significantly less frequently if the patient’s reason for seeing the doctor was another than purely somatic issues. The patients gave the aspects of *Patient’s opportunity to describe ones agenda* and *Patient being listened to without interruption* as well as *satisfaction* the highest possible rating significantly less frequently if they had another reason for the visit than something purely somatic (Table 4).

**Table 4:** Patient and doctor ratings for consultations with either somatic or other reasons for visit

Patient-centred aspect	Reason for visit (somatic/ other)	Total (no) of patient responses. Percentage for highest possible estimations, 95% confidence interval (CI)	Difference between patient responses *P*-value CHI 2-test	Total (no) of doctor responses. Percentage for highest possible estimations	Difference between doctor responses *P*-value CHI 2-test	Difference between doctor responses *P*-value F-test ICC = 0.1	Maximum ICC (doctors) for *P*-value ≤0.05 F-test
*1. The patient was provided an opportunity to describe their discomforts, ideas, concerns, and expectations*	Somatic	(158) 76.6% (CI 70.0–83.2)	0.016	(131) 73.3%	< 0.001	< 0.001	0.26
	Other	(43) 58.1% (CI 43.4–72.8)		(49) 36.7%			
*2.The doctor listened without interrupting*	Somatic	(161) 85.1% (CI 79.6–90.6)	0.047	(131) 74.8%	< 0.001	0.004	0.20
	Other	(43) 72.1% (CI 58.7–85.5)		(49) 42.9%			
*3. The patient was taken seriously*	Somatic	(160) 82.5% (CI 76.6–88.4)	0.605	(131) 78.6%	< 0.001	0.041	0.11
	Other	(43) 79.1% (CI 66.9–91.3)		(49) 57.1%			
*4. The patient was provided with information about the doctor’s assessment*	Somatic	(156) 77.6% (CI 71.1–84.1)	0.407	(130) 77.7%	0.003	0.069	0.07
	Other	(42) 71.4% (CI 57.7–85.1)		(48) 58.3%			
*5. The patient had their questions answered*	Somatic	(158) 71.5% (CI 64.5–78.5)	0.270	(131) 74.8%	< 0.001	0.030	0.14
	Other	(43) 62.2% (CI 48.4–77.2)		(48) 50.0%			
*6. The patient was provided an opportunity to participate in decisions concerning their ongoing care*	Somatic	(153) 64.1% (CI 56.5–71.1)	0.516	(131) 71.0%	< 0.001	0.002	0.37
	Other	(41) 58.5% (CI 43.3–73.6)		(47) 36.2%			
*7. Overall satisfaction*	Somatic	(160) 81.9% (CI 75.9–87.9)	0.040	(131) 79.4%	0.002	0.044	0.11
	Other	(43) 67.4% (CI 53.4–81.4)		(48) 58.3%			

Total number of patient and doctor responses and percentage of responses with highest possible estimation in connection with general practice consultation. Other reasons for visit than somatic consisted in mental, administrative/ certification (mainly sick-leave certificates), mixed or unspecific. The internal number of drop-outs varied between 19 and 29 for patients and 8 and 10 for the doctors

The number of reasons for the visit also had an impact on the ratings. It had a greater negative impact on the patients’ estimations than on the doctors’. Patients with two–six reasons for their visit gave the highest possible estimation on three of the aspects significantly less frequently than those with just one reason for the visit: *Patient listened to without interruption* [71.1% (CI: 57.9–84.3)] versus [85.5% (CI: 80.0–91.0)]*, Patient having their questions answered* [56.8% (CI: 42.2–71.4)] versus [73.2% (CI: 66.3–80.1)] and *Overall satisfaction* [64.4% (CI: 50.4–78.4)] versus [82.9% (CI: 77.0–88.8)]. The doctors’ estimations were similar regardless of number of reasons.

For patients born outside of Europe, the proportion of highest possible estimations on two of the aspects was significantly smaller than for patients born in Sweden: *Patient being taken seriously* [68% (CI: 55.1–80.9)] versus [84.3% (CI: 78.3–90.3)] and *Patient being provided with information on the doctor’s assessment* [65.3% (CI: 52.0–78.6)] versus [79.4% (CI: 72.6–86.2)]. Apart from the patient’s origin, no other examined background factor (age, sex, or level of education) had any impact on the patients’ estimations.

In the 64 matched questionnaire pairs where both doctors and patients reported the patient’s reason for visit, 87.7% of the patient–doctor pairs reported the same or not clearly contradictory reasons for visit. For 7.7% of the consultations, reasons for visit were reported in a way that made it impossible to determine if they were consistent or contradictory, whereas for 4.6% of the consultations patients and doctors reported completely different reasons for the encounter.

## Discussion

There are two main findings in this study. The first one concerns how the reason for the patient’s visit influenced the doctors’ estimations of their patient centredness – an aspect that has not previously been studied. Doctors estimated their communication to be patient centred significantly less frequently if patients had others reasons for their visits than purely somatic ones. This finding is supported by how patients perceived the consultation when they visited the doctor for other than purely somatic reasons: they less frequently gave the highest possible estimation on the aspects of soliciting the patient’s agenda, being listened to, and overall satisfaction.

The other main finding is that patients who had more than one reason for their visit indicated that they were not listened to without interruption, did not have their questions answered, and were not satisfied to the same extent as those with one reason for their visit. The doctors’ responses did not reflect that they perceived any of these differences in these consultations. This indicates that doctors fail to be patient centred, but also fail to notice this failure, when patients have two or more reasons for their visit.

In general, the patients rated their overall satisfaction higher than their opportunity to participate in the decisions about their care, and *shared decision-making* was the patient-centred aspect that was least present according to both doctors and patients.

### Reason for visit as a challenge to the doctor’s level of patient-centeredness

The results of our study suggest that it is mainly the nature of the patient’s reason for visit and not the patient’s background factors that challenge the doctor’s inclination to listen, explore, and involve the patient in the consultation. There were fewer top ratings of all patient-centred aspects in relation to “other reasons for visit than purely somatic”. This included mental illness, need of certificate (mainly for sick leave), other administrative tasks, a mix of somatic, mental and/or administrative reasons, or unspecific reasons. To find out if the association identified in this sample is especially connected to mental illness or to administrative tasks, for example the issuing of sick leave certificates, *or* if the association occurs whenever the patient is not seeing the doctor for a somatic problem, further studies need to be made.

### The “difficult” patient?

The findings can, however, be compared to predictors of patients being perceived as “difficult” by doctors. Factors like vague, complex and ambiguous medical problems, or psychiatric disorders such as clinical depression and anxiety, are more frequent in patients considered to be difficult [12, 28–31]. In fact, the probability of being considered as difficult increases with the number of psychiatric diagnoses, but it does not increase with the number of somatic diagnoses [32].

The task of issuing sickness certificates is also considered as problematic by doctors in general and general physicians in particular [33]. Patients regarded as difficult are more likely to have worse symptoms after their visit and are less content with their physician [29]. There is, consequently, reason to believe that when challenged with the complexity of certain medical problems, the doctors in this study are less inclined to use the patient-centred tools.

The experience of this group of patients could perhaps also reflect that the doctor is feeling too stressed to listen without interrupting or to solicit the patients agenda when faced with not purely somatic questions. Feeling inadequate in how to assess and help the patient with these questions being one possible explanation, too little time for a more complex assessment being another. Stressing the consultation has adverse consequences, however. Studies suggest that patients often need little time to tell their story [34], that the doctor misses important clinical information when patients are interrupted [35], and that when the patient’s agenda remains unveiled the risk for adverse effects increases [6–8].

### The patient with a list

The phenomenon that patients bring up several issues in the primary-care meeting is often brought up as a problem in consultation workshops, as the physicians experience that they have too little time to focus on more than one issue and that the consultation gets unfocused and messy. In this study, the patients with more than one reason for their visit were a bit less satisfied and experienced a lower frequency of being listened to and having their questions answered than those with one reason for their visit. One might suspect that patients expect to be able to bring up more than one problem when seeing the doctor, whereas primary care physicians tend to limit their task to one issue per visit. Addressing this early on in the consultation and making a plan for when to deal with the problems yet to be solved can perhaps be one way to reduce patient disappointment. Giving the doctors more time for consultation, thus enabling assessment of more than one medical problem per visit, could be another.

### Shared decision-making as a process and means to increase patient involvement

From previous studies we know that physicians in Sweden and other OECD countries in general lack in involving their patients in the decisions concerning their own care [1]. The findings in this study confirm this. An involving approach requires of the doctor to be educated in alternative strategies to handle a medical problem, including pros and cons with the different measures. The findings raise the question why doctors are better at listening to their patients than involving them in the decision-making. Listening can be seen as an independent part of the consultation, the beginning of the decision-making process. Involving the patient in decisions, on the other hand, is based on earlier steps such as soliciting and answering the patient’s questions, soliciting the patient’s preferences and giving the patient the relevant individualised information. Seeing the decision-making as a process instead of an event is in accordance with the patients’ perspectives on patient involvement, according to previous studies [36].

The new Patient Act that was introduced in Sweden in 2015 has the main purpose to increase focus on patient autonomy and strengthen patients’ involvement in their own care. A follow-up report from the Swedish Agency for Health and Care Services Analysis found that patient involvement has in fact decreased since the law was enacted [16]. In the report it is suggested that the patient’s legal position has to be fortified and that healthcare providers need information and education on the legislation. The findings from this study suggest that healthcare providers need specific training in the situations where patient involvement is compromised, such as consultations with psychiatric themes, complex reasons for visit, more than one reason for visit, medical uncertainty, and consultations with patient cases, generally or by the individual physician, perceived as difficult.

### Strengths and limitations

This study can, due to its exploratory character, contribute to generating new hypotheses regarding patient-centred aspects in general practice consultations. The focus on reasons for the visit contributes with data on a previously underexplored aspect of patient-centred care.

The fact that the study is limited in number of participants and restricted to only one primary care centre limits the generalisability of the results. However, the results of this study can be compared to results from the Swedish National Patient Questionnaire survey [37]. In the survey, patients rated their experience on items such as information, respect, and involvement in care at Swedish primary care centres. The primary care centre in our study was, in the national survey, rated marginally lower on these items in 2016 compared to the results from Swedish primary care centres in general. This suggests that the results could be interpreted as relevant to primary care in Sweden and similar settings elsewhere.

The choice to include the patient perspective instead of having an observer or taping the consultations is supported by previous findings in a number of studies that show that patient questionnaires or other measures to capture the patients’ perspective are better at predicting outcomes than observations or physicians’ perceptions [13, 14].

To give the patients and doctors an opportunity to reflect on the consultation in immediate connection to their meeting was essential, as it would be hard for the respondents to remember the course of events if asked later. However, collecting the questionnaires in such tight connection to the visit, and in the midst of the healthcare practice, might have affected the patients to be less confident that their responses would not reach the doctor and that their anonymity would not be compromised – in spite of the study information and the present researchers’ assurance that no healthcare providers would get access to their responses. This could have had an effect on how critical the patients dared to be. Hence, we cannot exclude that those who were most displeased were overrepresented among non-participants.

There is a risk that the respondents who were content with their encounter tended to choose the “I agree completely” response option for all the aspects without paying much attention to the actual statement (halo effect). It is also easier for the respondents to think in terms of *good/bad* instead of *was this done or not*? Variation in wording of the statements they responded to, thus forcing them to give each individual question some more consideration, might have limited the effect of respondents answering in a non-considered way.

We chose to dichotomise the four response options to the statements on aspects of patient centredness and satisfaction by comparing the alternative “I agree completely” to the other three less concurring options: “I agree to a large extent”, “I disagree to a large extent”, and “I disagree completely” [1, 38, 39]. While acknowledging that dichotomising into “agrees” and “disagrees”, with two response options in each group, would not identify any differences whatsoever between patients and healthcare providers, our rationale for our way of slicing the cake was that if an encounter were fully satisfactory in terms of patient centredness, then this would have been reflected in “I agree completely” responses. A response to the effect that the respondent largely agrees nevertheless indicates that there was something that could have been handled better.

Ideally, the results would have been corrected for intra class correlations (ICC) estimated from this sample, and the background factors of the physicians would have been included in the analysis of factors interfering with the patient-centred aspects. Due to consideration for the participating physicians’ wish that neither the base-line survey nor the questionnaires should be coded to the individual physician for confidentiality reasons, these analyses could not be performed. Instead additional sensitivity analyses were performed for the observations from the physicians’ questionnaires for those results where significant differences were found. In this study we assumed no clustering for the patients and a level of clustering corresponding to an ICC of 0.1 for doctors. The 188 doctor responses are considered to be dependent due to the fact that they are answered by only16 doctors, each doctor filling out more than one questionnaire. We were not able to find comparable studies reporting ICC values. For that reason we also present the maximum ICC for each comparison of doctor responses where the differences are still significant at a 5%-level.

## Conclusion

In the responses to the present survey on aspects of patient-centred consultations in primary care, *shared decision-making* came out worst in both groups, the highest possible estimation being notably less frequent than full patient and doctor satisfaction with their encounters. The results suggest that factors such as the nature of the reason for the visit and the number of reasons for the visit might challenge the doctors’ inclination to communicate in a patient-centred manner, with a native patient presenting a single biomedical reason for visit as seemingly optimal. Further studies are needed to explore which types of reasons for encounter that poses the greatest challenges for the doctors to retain a patient-centred approach.

### What is already known on this topic

Patients want a patient-centred approach and there is currently room for improvement when it comes to making the patient informed and involved in the decisions about their care.

### What the study adds

Factors like the nature of the patient’s reason for encounter and number of reasons for encounter might affect the doctors’ inclination to communicate in a patient-centred manner.

## Additional files

Additional file 1: The patient-centred consultation model of the physicians program at Karolinska Institutet. (DOCX 15 kb)

Additional file 2: The patient questionnaire. (DOCX 49 kb)

Additional file 3: The doctor questionnaire. (DOCX 48 kb)

## Abbreviations

CI: Confidence Interval
ICC: Intra class correlation
ICE: Ideas, concerns and expectations
OECD: The organisation for economic Co-operation and development
WHO: World health organisation

## Authors’ information

HB is a general physician at Gustavsberg Primary Care Centre, clinical teacher at the physicians program at Karolinska Institutet and PhD-student at Stockholm Centre for Medical Ethics (CHE). NL is senior professor, GH professor and NJ associate professor in medical ethics and supervisors to HB at CHE in the Department of Learning, Informatics, Management and Medical Ethics (LIME) at Karolinska Institutet. DO is statistician at the Unit of Medical Statistics, LIME, Karolinska Institutet.
