# Supplementary material for: Challenges to patient centredness – a comparison of patient and doctor experiences from primary care
Source: BMC Fam Pract. 2019 Jun 15;20:83. doi: 10.1186/s12875-019-0959-y (PMC6570949; doi:10.1186/s12875-019-0959-y)
Supplement: Supplementary file 1 — The patient-centred consultation model of the physicians program at Karolinska Institutet. (DOCX 15 kb) [file 12875_2019_959_MOESM1_ESM.docx]

**The patient-centred consultation model of the physicians program at Karolinska Institutet**

In the patient-centred consultation model taught to students and supervisors in the physicians program at Karolinska Institutet, the items are operationalised chronologically in three parts: the patient’s part, the doctor’s part and the shared part.

The patient’s part

- The doctor initiates with an open invitation and provides the patient with an opportunity to present their reason for their visit and spontaneous descriptions of problems without interruption.
- The doctor facilitates the patient’s recounting through summaries and emotional validation.
- The doctor pays a special attention to the patient’s questions, clarified through the patient’s own ideas about the problem, fears, concerns, and expectations/ wishes (ICE) regarding the visit and asks for these when unspoken by the patient.
- The doctor summarises the patient’s narration and agenda (reason for visit and ICE).

The doctor’s part

- The doctor further explores the patient’s problem in a focused medical interview and physical examination and involves the patient in the meaning of the questions and examinations when not obvious.

The shared part

- The doctor summarises and addresses the patient’s initial questions and agenda.
- The doctor explains and gives information on assessment and what it is based on.
- The doctor clarifies and answers any new questions, in order to make joint decisions with the patient and find common ground in how to handle the problems presented.
- The doctor and patient are both involved in making a plan for ongoing care and contingency and the patient is asked to summarise their “take home-message”.
