# Supplementary material for: Challenges to patient centredness – a comparison of patient and doctor experiences from primary care
Source: BMC Fam Pract. 2019 Jun 15;20:83. doi: 10.1186/s12875-019-0959-y (PMC6570949; doi:10.1186/s12875-019-0959-y)
Supplement: Supplementary file 2 — The patient questionnaire. (DOCX 49 kb) [file 12875_2019_959_MOESM2_ESM.docx]

**1. What was the main reason for your visit to the primary health care centre? What did you wish to gain from your visit?**

**2. Where there also other reasons for your visit? If so, which one/s?**

Please respond to the following claims by marking with a cross the response alternative that best agrees with your perception:

**3. You described your own ideas regarding your ailment/problem, your concerns and what**

**you wished for/expected of the visit**

I agree completely I agree to a large extent I disagree to a large extent I disagree completely

**4. The caregiver listened to you without interrupting**

I agree completely I agree to a large extent I disagree to a large extent I disagree completely

**5. You experienced that you were taken seriously when you told about your ailments/problems**

I agree completely I agree to a large extent I disagree to a large extent I disagree completely

**6. You were informed about the caregiver’s assessment on your need for measures to be taken**

I agree completely I agree to a large extent I disagree to a large extent I disagree completely

**7. You got your questions answered**

I agree completely I agree to a large extent I disagree to a large extent I disagree completely

**8. You were invited to participate in the decision-making regarding your care (examination/treatment)**

I agree completely I agree to a large extent I disagree to a large extent I disagree completely

**9. You are satisfied with your visit**

I agree completely I agree to a large extent I disagree to a large extent I disagree completely

Your age: _______

Your sex: Woman Man

Country of birth: Sweden Other European country Born outside Europe

Highest level of education (elementary school, high school/gymnasium, professional school, university):

Would you like to comment? Please use the flipside of the paper!

*Thanks for your participation!*
