# Supplementary material for: Challenges to patient centredness – a comparison of patient and doctor experiences from primary care
Source: BMC Fam Pract. 2019 Jun 15;20:83. doi: 10.1186/s12875-019-0959-y (PMC6570949; doi:10.1186/s12875-019-0959-y)
Supplement: Supplementary file 3 — The doctor questionnaire. (DOCX 48 kb) [file 12875_2019_959_MOESM3_ESM.docx]

**1. What was the main reason for the patients visit? What did the patient wish to gain from the visit?**

**2. Where there also other reasons for the patient’s visit? If so, which one/s?**

Please respond to the following claims by marking with a cross the response alternative that best agrees with your perception:

**3. The patient described their own ideas regarding their ailment/problem, their concerns and what**

**he/she wished for/expected of the visit**

I agree completely I agree to a large extent I disagree to a large extent I disagree completely

**4. I listened to the patient without interrupting**

I agree completely I agree to a large extent I disagree to a large extent I disagree completely

**5. I took what the patient told me seriously**

I agree completely I agree to a large extent I disagree to a large extent I disagree completely

**6. The patient was informed about my assessment on the need for measures to be taken**

I agree completely I agree to a large extent I disagree to a large extent I disagree completely

**7. The patient got their questions answered**

I agree completely I agree to a large extent I disagree to a large extent I disagree completely

**8. The patient was invited to participate in the decision-making regarding their examination/treatment**

I agree completely I agree to a large extent I disagree to a large extent I disagree completely

**9. I am satisfied with my performance during the visit**

I agree completely I agree to a large extent I disagree to a large extent I disagree completely

Your profession: Physician Nurse Other

Your sex: Woman Man

Would you like to comment? Please use the flipside of the paper!

*Thanks for your participation!*
